# Efficiency of ultrasound for degradation of an anionic surfactant from water: Surfactant determination using methylene blue active substances method

**DOI:** 10.1016/j.mex.2019.03.028

**Published:** 2019-04-17

**Authors:** Mohammad Hadi Dehghani, Ahmad Zarei, Mahmood Yousefi

**Affiliations:** a Department of Environmental Health Engineering, School of Public Health, Tehran University of Medical Sciences, Tehran, Iran; b Institute for Environmental Research, Center for Solid Waste Research, Tehran University of Medical Sciences, Tehran, Iran; c Department of Environmental Health Engineering, School of Public Health, Gonabad University of Medical Sciences, Gonabad, Iran

**Keywords:** Application of efficiency of ultrasound frequency for degradation of an anionic surfactant from water using methylene blue active substances method, Sonoreactor, Linear alkylbenzene sulfonates, Methylene blue active substances

## Abstract

The removal of a surfactant from wastewater is usually difficult due to its toxicity and low biodegradability. The aim of this study was to apply sonoreactor for degradation of an anionic surfactant from aqueous solution. An ultrasonic bath with frequency of 130 kHz was used to investigate the effects of different operational parameters such as sonication time, initial concentration and power. In this study, experiments of linear alkylbenzene sulfonates solution were performed using methylene blue active substances method. Experiments were performed at initial concentrations of 0.2 , 0.5 , 0.8  and 1 mg/L, frequency of 130 kHz, acoustic powers value of 400 and 500 W, temperature of 18–20 °C and pH value of 6.8–7. This study showed that linear alkylbenzene sulfonates degradation rate was found to increase with increasing sonication time and power. In addition, as the concentration increased, the linear alkylbenzene sulfonates degradation rate decreased in the ultrasonic reactor.

• Surfactants are one of the largest groups of pollutants which exist in almost all urban and many industrial wastewaters.
• Ultrasonic reactors alone may not be useful for reducing completely complex wastewaters of high surfactant load.
• Application of ultrasonic reactors in combination with other treatment processes including Ozone, UV irradiation, chlorination, Fenton, nanoparticles and H_2_O_2_ could be used as a pre-treatment unit in a sequential chemical and biological treatment process.

## Graphical abstract

**Figure:**
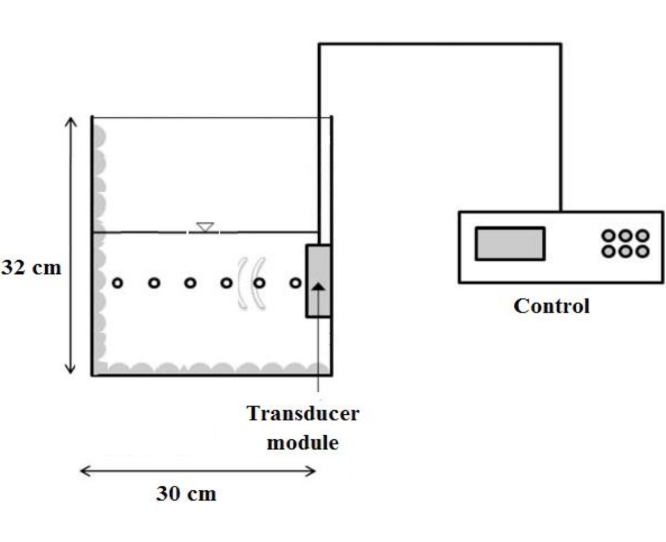

**Specifications Table**

**Table:** 

Subject area:	Environmental Sciences
More specific subject area:	Degradation of an anionic surfactant
Methods name:	Application of efficiency of ultrasound frequency for degradation of an anionic surfactant from water using methylene blue active substances method
Name and reference of original method:	S.H. Venhuis and M.Mehrvar, Health effects, environmental impacts, and photochemical degradation of selected surfactants in water. Int. J. Photoenergy, 6 (2004)115–125
Resource availability:	The data are available in this article.

## Method details

In recent decades, with the rapid development of urbanization and industry, the quantity wastewater generation has increased dramatically, while the increased wastewater endanger many surface and groundwater resources, and becomes the environmental issue that communities have to overcome [[1], [2], [3], [4], [5], [6]]. Therefore, more and more environmentalists are getting interested in the application of efficient wastewater treatment methods, in particular, surfactants have considerable effects on water ecosystems and consequently human health. Surfactants are one of the most important pollutants which exist in almost all urban and many industrial wastewaters. Large quantities of surfactants cause many environmental damages by entering the water bodies and soil. Anionic surfactants are the most widely used in household detergents, consumer products and industries [[7], [8], [9]].

Anionic surfactants especially linear alkylbenzene sulfonates (LAS), cause biochemical, pathological, physiological, and other impacts on aquatic/terrestrial ecosystems [[7], [8], [9], [10]]. In aquatic ecosystem, they have effects such as chlorophyll damage, cell death and growth reduction [11,12]. Fairchild et al. have also reported that linear alkylbenzene sulfonates concentration of 0.36 mg/L had no impact on microorganisms [11]. Venhuis and Mehrvar have estimated that 0.02–1.0 mg/L linear alkylbenzene sulfonates in aquatic ecosystem can damage fish and mussel larva and 40–60 mg LAS/kg dry weight of sludge interfere with the reproduction of soil invertebrates [12]. Mehrvar evaluated acute effects of linear alkylbenzene sulfonates on plankton, bacteria, crustaceans, earthworms, flagellates, ciliates and enchytracids [12]. Vande Plassche et al. reported that a concentration of 0.25 mg/L of linear alkylbenzene sulfonates have no effect on aquatic populations [13].

A large number of surfactants, including the linear alkylbenzene sulfonates are not easily biodegradable. Sonochemical reactor (or Sonoreactor) has been investigated as a viable advanced oxidation processes for the removal of surfactants in the past one and half decade [[14], [15], [16], [17], [18], [19]]. This technology is environmentally friendly. Ultrasonic technology has the advantages such as no chemical use, easily installation and operation, no sludge, no by-products, requiring small area, low maintenance and operation costs [19,20].

In recent years, considerable interest has been shown on the effectiveness of ultrasonic reactor as a novel technology for the degradation of contaminants from water and wastewater [[21], [23], [24]].

Ultrasonic waves can induce mechanical, thermal and chemical effects in environment due to the pressure gradient and cavitations. Producing bubbles [25] depends on the acoustic pressure differences. Gas bubbles can be reduced or destroyed by increasing water pressure. All solutions contain significant amounts of gas bubbles. As the result of mechanical quakes, these bubbles reach to certain diameter in certain specific wavelengths, ultrasonic waves (6 μm in diameter, 1 MHz frequency) and cause characteristics in their resonance in such a way that amplitude oscillations will be bigger. On the other hand, due to severe fluctuations and high pressure of gas inside the bubbles, a phenomenon similar to the gas ionization produce free radicals and cause the higher density of radicals around the water molecules [[26], [27], [28], [29], [30], [31], [32]].

Generally, generation of free radicals during sonolysis is described by the following equations in the presence of dissolved oxygen in aqueous solution [17]:

(1) H_2_O → H^•^ + ^•^OH

(2) O_2_ → 2O^•^

(3) ^•^OH + ^•^OH → H_2_O + O^•^

(4) ^•^OH + ^•^OH → H_2_ + O_2_

(5) ^•^OH (*aq*) + ^•^OH (*aq*) → H_2_O_2_

(6) ^•^OH + H_2_O → H_2_O_2_ + H^•^

(7) H^•^ + ^•^OH → H_2_O

(8) H^•^ + H^•^ → H_2_

(9) O^•^ + O^•^ → O_2_

(10) O^•^ + H_2_O → 2^•^OH

(11) O_2_ → O^•^ + O^•^

(12) O_2_ + O^•^ → O_3_

(13) H^•^ + O_2_ → HO_2_^•^

(14) HO_2_^•^ + H^•^ → H_2_O_2_

(15) H_2_O^•^ + H_2_O^•^ → H_2_O_2_ + H_2_

(16) H_2_O^•^ → ^•^OH + 1/2 H_2_

The main objective of this article was to evaluate the effect of ultrasonic reactor as an advanced oxidation process and to provide a greater knowledge of the fundamentals of sonotreatment of anionic surfactants solution via acoustic bubble process. Also, in the present work, the degradation rate of linear alkylbenzene sulfonate is evaluated with emphasis on the effect of sonocation time, initial concentration and acoustic power. In this study we compared the two applied powers of 400  and 500 W for the degradation of LAS from aqueous solution. In this study, surfactant was determined using methylene blue active substances method. The method is based on the formation of an ionic pair between the anionic surfactants, AS, and the methylene blue, MB. However, although this method is standard and have high accuracy and precision but takes a relatively long time to do and also requires great amounts of chloroform and sample.

## Materials and methods

### Reagents, reactor set up, experiments and analysis

**Fig. 1:**
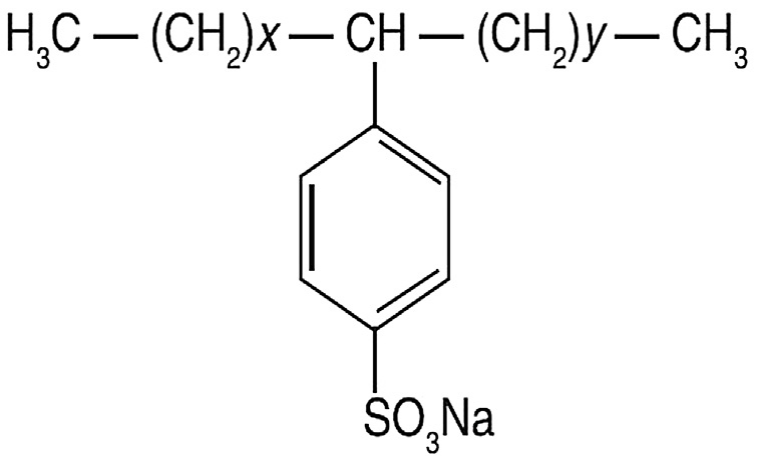
Structure of LAS [33].

The chemicals used in this study including chloroform, sulphuric acid, sodium hydroxide, sodium dihydrogen phosphate monohydrate, methylene blue, phenolphthalein, and LAS were supplied by Merck Company. All chemicals were used as received. Structure of LAS is shown in Fig. 1.

**Fig. 2:**
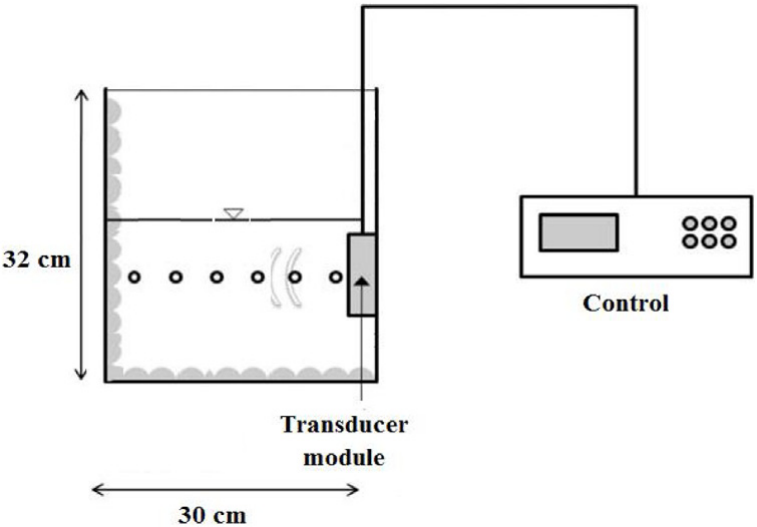
Set up of sonoreactor for degradation of LAS.

**Fig. 3:**
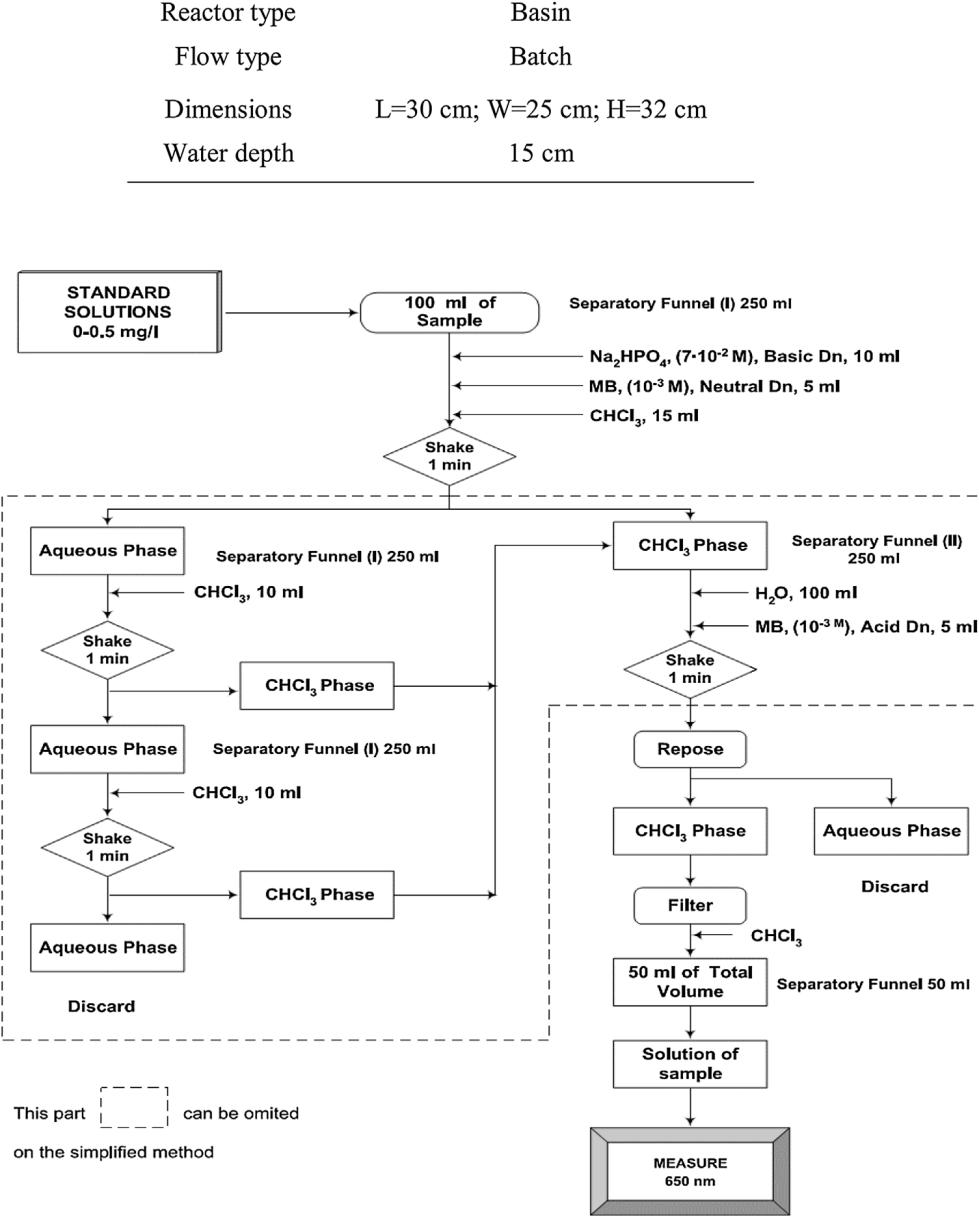
Diagram presenting the steps of the procedure for LAS determination [34].

A batch reactor was used for the experiments, which was closed during ultrasonic irradiation. Sonication experiments were conducted in an ultrasonic reactor and an ultrasonic transducer operating at 130 kHz. A set up of the reactor used in this study is illustrated in Fig. 2. Characteristics of sonochemical reactor used in the experiments is given in Table 1. The concentration of LAS in the aquatic phase was determined using method “5540 C Anionic Surfactants as Methylene blue active substances (MBAS)” described in the Standard Methods for the Examination of Water and Wastewater. Samples were withdrawn from reactors at specified times during sonication. Duplicate degradation runs were performed to verify results at selected sonication times. It comprises of 3 successive extractions from acid aqueous solution containing excess methylene blue into chloroform (CHCl_3_), followed by an aqueous backwash and measurement of the intensity of blue color in the CHCl_3_ by using a UV/Vis spectrophotometer at wavelength 652 nm. Diagram presenting the steps of the procedure for LAS measurement is shown in Fig. 3.

**Table 1:** Characteristics of sonochemical reactor used in the experiments.

Parameters	Characteristics
Frequency	130 kHz
Power	400 and 500 W
Reactor type	Basin
Flow type	Batch
Dimensions	L = 30 cm; W = 30 cm; H = 32 cm
Water depth	15 cm

Minimum detectable quantity is about 10 mg MBAS (calculated as LAS). Regarding precision and bias, a relative standard deviation of 9.7% and a relative error of 1.5% was obtained. All the analyses were performed according to the procedures outlined in standard methods for the examination of water and wastewater.

### QC and QA measures

The recovery of LAS in aqueous samples was measured by adding a predetermined amount of LAS concentration to the synthetic substrate. The results were calculated using the following expression:

Recovery percentage = (mg/L obtained/mg/L theoretical) × 100

The recovery percentage of this method was 96%.

## Results and discussion

In this study, LAS was sonodegraded at different contact times including 20, 30, 40, 50, 60, 70, 80, 90, 100, 110 and 120 min. Also, sonodegradation experiments of LAS were carried out in the presence of various concentrations to observe if there was any effect on the degradation of LAS. Sonodegradation of LAS was performed at initial concentrations of 0.2 , 0.5 , 0.8  and 1 mg/L, acoustic frequency of 130 kHz, pH 6.8–7.0 and applied power of 400 and 500 W. The temperature was maintained at 18–20 °C.

### Influence of initial concentration

**Fig. 4:**
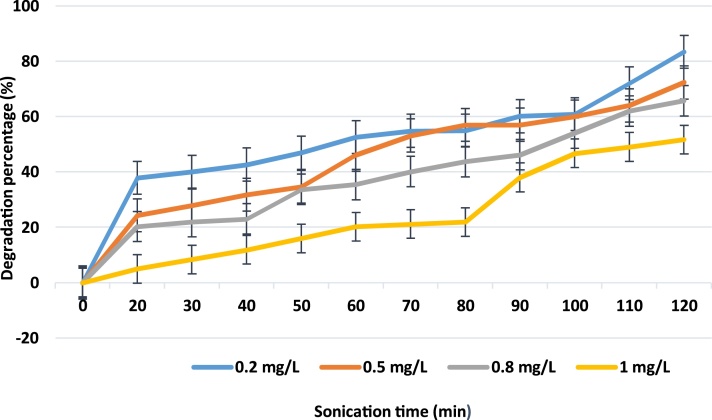
Degradation percentage of surfactant vs. treatment time for different concentrations (400 W).

**Fig. 5:**
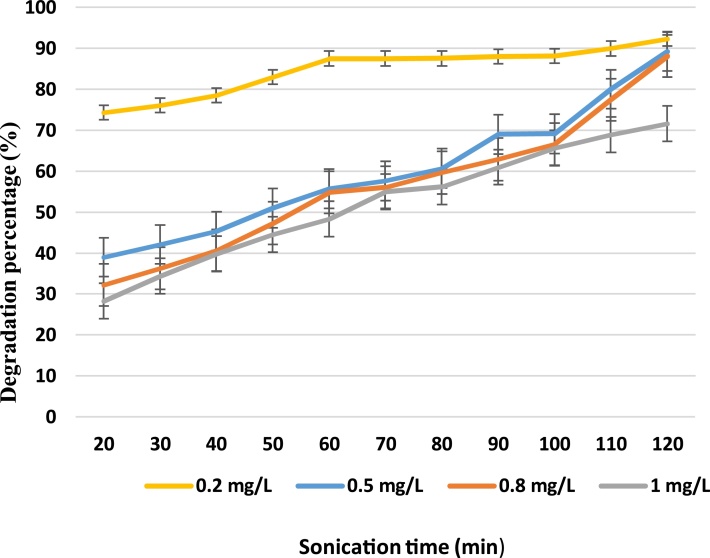
Degradation percentage of surfactant vs. treatment time for different concentrations (500 W).

Experiments were conducted in various times to see if there was any synergistic effect on the degradation of LAS. Increasing the concentration from 0.2 to 1 mg/L showed a decrease in degradation of LAS. Experiments showed that in sonochemical reactor, about 83.30, 72.28, 65.69 and 51.70% degradation of LAS occurred during 120 min but only 37.84, 24.27, 20.25 and 5.00% degradation of LAS was observed within 20 min as shown in Fig. 4 (400 W). Also, experiments showed that in this reactor, about 92.35, 89.21, 88.11 and 71.60% degradation of surfactant occurred during 120 min and 74.36, 39.00, 32.28 and 28.29% degradation of surfactant was observed after 20 min as shown in Fig. 5 (500 W). Therefore, results obtained from the sonochemical degradation of LAS at various concentrations indicated that removal rates were found to decrease with increasing LAS concentration.

Using one-way ANOVA, we found statistically significant differences in different concentrations (P value < 0.001. Also, statistical analysis using Post-hoc test showed that there was significant difference between 0.2 mg/L and 1 mg/L (P value < 0.001). But there are no significant differences for other concentrations, such as 0.2–0.5 mg/L, 0.5–0.8 mg/L and 0.8–1 mg/L (P value > 0.001). Using multiple regression indicated that there were linear relationships between sonication time and initial concentration. On the other hand, the linear relationships equations for degradation percentage are as follows:

Degradation = 23.096 + 0.426 Time

### Influence of sonication time

In order to observe the effect of sonication time on the LAS degradation rate during treatment, sonodegradation or sonication time for aqueous LAS concentrations was performed in twelve intervals. As clearly seen, by increasing the sonication time, considerable levels of LAS degradation can be expected after 120 min. It was observed that the degradation efficiency of acoustic frequency was increased when sonication time was increased. Therefore, the statistical study using Pearson correlation tests indicated that when sonication time is increased, there is an increase in removal percentage (P value < 0.001, r = 0.638). This effect is due to the increased oppurtunity of the LAS solution and the acoustic cavitation process for reaction as the time of sonication increases [17,18].

### Influence of initial pH value

The experiments were performed at pH 6.8-7. This study showed that the pH has no significant influence on the degradation of LAS using ultrasonic irradiation. Some other studies showed that the degradation rate of contaminants is decreased by increasing the pH of the solution [27,28,36].

### Influence of acoustic power

This research showed that the degradation rate is increased with an increase of acoustic power, because acoustic power may lead to more extensive acoustic cavitation. The effect of acoustic power on the sono-degradation of LAS may be described in terms of sono-chemical reactivity. High levels of acoustic power increase the number of cavitational events and consequently the opportunities for free radicals to be generated enhancing degradation [19,25,29,37]. This is in agreement with the results reported by other studies [23,38]. Manousaki et al. studied the degradation feasibility of sodium dodecylbenzene sulfonate in aqueous solution by ultrasonic irradiation. Various parameters including initial concentrations of (15, 30 and 100 mg/L), ultrasonic frequencies (20 and 80 kHz) and applied power values (45, 75 and 150 W) were considered. At the conditions in question, sodium dodecylbenzene sulfonate degradation alleviated with decreasing initial pollutant concentration and deceasing power. Totally, the use of ultrasound enhanced the aerobic degradability of the substrate in question [23]. Lijun et al. studied linear alkyl benzene sulphonate (LAS) degradation by immobilized *Pseudomonas aeruginosa* under low intensity ultrasound. They found that ultrasonic irradiation promote the LAS biodegradation. With the increase of the LAS concentration, the degradation rate decreased [39]. In a study Naldoni et al. mineralized surfactants using ultrasound and the advanced Fenton process. The application of 20 kHz ultrasound leads to extensive mineralization of sodium dodecylbenzene sulfonate (DBS) and dodecyl pyridinium chloride (DPC) as determined by total organic carbon (TOC) measurements. Bin Abu Hassan et al. investigated the effect of homogeneous catalyst for the degradation of Sodium Dodecylbenzene Sulfonate (SDBS) in water by means of ultrasonic irradiation. In their study, ultrasound increased the removal efficiency of SDBS [40].

## Conclusions

A sono-based treatment method has been used for the removal of LAS from aqueous dispersion. Results obtained from this study showed that ultrasonic reactors at a frequency 130 kHz and powers 400 and 500 W was capable to degrade LAS from aqueous synthetic solutions. Potential of ultrasonic reactors for LAS degradation is evaluated with emphasis on the effect of sonication time and initial concentration. Experiments showed that sonication time is one of the most important parameters for LAS degradation. Also, this study indicates that treatment efficiency increases with the decreasing concentration. However, ultrasonic reactors alone may not be highly efficient for reducing complex wastewaters with high surfactant loads. Thus, the application of ultrasonic reactors in combination with other treatment processes including Ozone, UV irradiation, chlorination, Fenton, nanoparticles and H_2_O_2_ could be used as a pre-treatment unit in sequential chemical and biological treatment processess.
